# News and Events of the Week

**Published:** 1910-03-05

**Authors:** 


### News and Events of the Week.

The eleventh annual Medical Congress of Russian practitioners will be held at St. Petersburg on April 21-28.

Dr. Robert Jones has been placed on the Commission of the Peace for the County of Essex by the Lord Chancellor, upon the nomination of the Lord Lieutenant.

The University of Pennsylvania has received the sum of $100,000, through the influence of a medical alumnus, to found a chair in the Medical Department not yet designated.

The annual meeting of the Association for the Prevention of Premature Burial was held at Anderton's Hotel, Fleet Street, on Thursday last week, the Right Hon. Sir Walter Foster in the chair.

A Bill to regulate the qualifications of trained nurses and to provide for their registration has been presented by Mr. Munro Ferguson (Leith Burghs), and appointed for a second reading.

At the last meeting of the Central Midwives Board the Secretary announced that a diploma of honour had been conferred on the Board by the Seventh National Congress of Midwives, held at Bologna last September.

The Philadelphia Polyclinic and College for Graduates in Medicine announces a twelve-week course of Tropical Medicine three times yearly. The sessions commence on October 1, January 1, and April 15. The fee for the course is $50.

The Right Hon. Sir Henry Roscoe, F.R.S., and Professor Sir William Ramsay, F.R.S., have been appointed to represent London University at the Centenary of the Königliche Friedrich-Wilhelms-Universität, to be celebrated in Berlin in October, 1910.

At the International Congress of Otology, to be held at Boston in 1912, a two hundred dollar prize, founded by Professor Cozzolino, of Naples, will be awarded for the best work on the treatment of progressive deafness or on a mechanical device for relieving it.

After a lapse of many weeks—the delay being due for the most part to the death of the Chairman, Lord Selby, and the General Election—the Royal Commission on Vivisection held a further meeting on Tuesday, Feb. 22, to consider the draft report of the Chairman.

Special sermons were preached in St. Mary's Church, Manchester, last Sunday, on behalf of the Manchester and Salford Medical Charities, by the Rector, the Rev. J. Waring. The collections realised the satisfactory total of £13, a substantial increase on last year.

Mr. S. G. Shattock, F.R.C.S., Curator of the Museum of St. Thomas's Hospital and Pathological Curator of the Museum of the Royal College of Surgeons, and Dr. J. H. Parsons, D.S.C., F.R.C.S., Lecturer on Physiological Optics at the College, have been appointed Fellows of University College, London.

The International Congress on the Administrative Sciences will take place at Brussels from July 27 to July 31 this year. The Belgian Government has officially invited foreign countries to send delegates to the Congress. The Hon. Secretary to the British Committee is Mr. G. Montagu Harris, Caxton House, Westminster, S.W.

The Prince and Princess of Wales have given their patronage to the Keats-Shelley *matinée* at the Haymarket Theatre on Friday, June 10, which is being organised to provide for the complete equipment of the Memorial House in Rome. Influential literary and social support is being given to the *matinée*, the special features of which will be announced later.

The New York medical supply depot of the United States Army was destroyed by fire on February 4, 1910. The loss is estimated to be between $500,000 and $1,000,000. The New York depot was the most important one owned by the Government for medical supplies of the Army, as through it went two-thirds of all the Army medical supplies.

The St. Pancras M.O.H. has informed the Public Health Committee that in London in 1908, as the number of deaths from puerperal fever was 137 and the number of cases was 228, the mortality was 60 per cent. It is possible, says the committee, that prompt treatment in hospital would reduce both the mortality and prevalence of the disease, and the amount of hospital accommodation necessary would be small.

The directors of the Lemco and Oxo Company (Liebig's Extract of Meat Company, Limited), acting under agreement with the directors of the Chartered Company, recently sent an expert to pay an extended visit to Rhodesia, and to select several large blocks of land for development as additional farms for the Lemco and Oxo Company. Already something like 500,000 acres have been purchased and the company has the option of extending. It is intended to commence developing these farms as soon as they have been surveyed.

Messrs. Puttick and Simpson have recently disposed of a collection of curious portraits of notable characters, including a series of prints of "Quacks, Empirics, and Eccentrics," among them being portraits of James Morrison, "The Hygiest," St. John Long, "as he appeared at his defence in the Old Bailey, February 19, 1831," a fancy portrait of Sanctorius "in his balance," Lord Barrymore, "the stroker," and Master Van Butchell, pupil of Dr. Hunter. An advertisement of Van Butchell was also included; it ran: "By the Grace of God we cure toothache in twenty minutes, without drawing it; likewise bad ruptures, very speedily, with fit bandages that do not torture. Master Van Butchell, surgeon for the teeth for more than thirty years. They that seek shall find."

Two medical missionaries are urgently needed, says the official organ of medical missions, one for Salt, Palestine, to take the place of Rev. Dr. R. B. Bryan, and the other for Mosul, in Turkish Arabia. At this latter place the promising work restarted by Dr. Griffith is, for the time being, suspended. It is hoped that negotiations, recently opened with the permission of the committee with a highly qualified Syrian doctor, may be successful, and that early this year the medical work may be reopened. But a European medical superintendent is an essential and urgent need. It may be well to mention, for the guidance of any qualified men who may offer for either of these posts, that the passing in French or Turkish of a very simple medical examination at the Constantinople Medical School is, by Turkish law, a necessary preliminary.

Last Saturday, at the Durham Assizes, Mr. C. G. B. Knapp, medical practitioner, was acquitted on a charge of performing an illegal operation upon Miss A. I. Handysides, at Gateshead, on January 13.

The annual general meeting of the Medical Graduates' College and Polyclinic will be held at 22 Chenies Street, London, W.C., on Friday, March 11, 1910, at 5.15 p.m. The attendance of all members and subscribers is particularly requested.

The Rochdale and District Medical Society has passed a resolution dealing with the position of Dr. Harris, against whom charges were made by the plaintiff in the recent dispute over a medical certificate heard at Manchester. The Society regrets that Dr. Harris is unable to reopen the case, and so to make all the facts public.

The Departmental Committee appointed to consider the working of the Midwives Act, 1902, recommend (paragraphs 71 and 77 of the report) that the Act of 1902 should be amended so as to give any medical practitioner summoned by a midwife in cases of emergency "a secure expectation of payment"; and that the Poor Law Authority should be responsible for the fee when the medical man cannot otherwise obtain payment, and should be empowered, if they think fit, to charge the fee paid as "relief on loan."

Dr. Gordon Craig contributes to the January number of the *Australasian Medical Gazette* a most interesting and readable article on the Mayo Brothers' Clinic at Rochester, with special reference to open ether anæsthesia. No fewer than 40,000 consecutive cases of anæsthesia under this method have been recorded at this clinic without a single fatality. To the February number of the *St. Thomas's Hospital Gazette* Mr. Makins also contributes an article on his impressions of America, in which his visit to the Rochester Clinic is described.

Mr. William Ray, B.Sc., Rhodes scholar and commoner of Magdalen College, has been elected to the Philip Walker studentship at Oxford. He is a graduate in medicine and science of the University of Adelaide, and came to Oxford in 1907. He has been engaged since then in research work in pathology, and in June 1909 gained the B.Sc. degree for a thesis on "Changes in the Leucocytes during Immunisation." He collaborated with Professor Dreyer in pathological investigation as to the phenomena of "Passive Immunity," and, as a Philip Walker student, will pursue these studies, and particularly the treatment of infectious diseases. The studentship is worth £200 a year for three years.

The following donations have been received for Professor Nuttall's appeal to buy a site and to build, equip, and conduct a field laboratory on the outskirts of Cambridge for research work in parasitology. The laboratory will be mainly for the study of protozoal and parasitic diseases. The laboratory and the funds are to become the property of the University: The Duke of Bedford, £100; Lord Rothschild, £100; Sir R. P. Cooper, £100; the Transvaal Government, £500; Colonial Office (Tropical Diseases Research Fund), £25; Dr. F. Darwin, F.R.S., £5 5s.; and others—total, £988 17s. £1,000 has been promised, anonymously, when the fund has reached £6,000. In addition, the Government of Cape Colony has placed £500 at the disposal of Professor Nuttall for investigating East Coast fever. By permission, part of this sum will be used for building the laboratory.

To show how the disease may be checked, the West Ham Town Council recently decided to hold a tuberculosis exhibition. The National Society for the Prevention of Consumption provided lecturers and exhibits. The Mayor of West Ham (Alderman G. J. Hosking) opened the exhibition on February 26 at the Public Hall, Canning Town. The exhibition consists of models, photographs, drawings, and demonstrations.

### COMING EVENTS OF THE WEEK.

**Monday, March 7.**—The Princess of Wales opens the new building of the Hospital for Invalid Gentlewomen, 19 Lisson Grove, 3.30.

Royal College of Surgeons: Professor R. H. Paramore's lecture on "The Functions of the Pelvic Floor Musculature," 5.

Medical Society: Dr. J. S. Risien Russell's Lettsomian Lecture on "The Cerebellum and its Affections," 9.

Royal Amateur Art Society: Annual Exhibition in aid of London charities, 1 Hamilton Place, Piccadilly, opens.

**Tuesday, March 8.**—Royal Institution: Prof. F. W. Mott's fifth lecture on "The Emotions and their Expression," 3.

Royal College of Physicians: Dr. J. S. Bolton's Goulstonian Lecture on "A Contribution to the Localisation of Cerebral Function, based on the Clinico-Pathological Study of Mental Disease," 5.

Pharmaceutical Society of Great Britain: Meeting, 8.

Annual meeting, British Lying-in Hospital, Endell Street, 5.

**Wednesday, March 9.**—Royal College of Surgeons: Professor C. Bolton on the "Pathology of Gastric Ulcer," 5.

Earl of Sandwich presides at annual meeting of Royal Free Hospital, 4.

Dinner, Balneological Section R.S.M., Paganis Restaurant, 7.

**Thursday, March 10.**—Royal College of Physicians: Professor W. Osler's Lumleian Lecture on "Angina Pectoris," 5.

Annual meeting, New Hospital for Women, 144 Euston Road, 4.

Annual meeting, Great North Central Hospital, Holloway Road, 5.

Annual meeting, French Hospital, 172 Shaftesbury Avenue, 6.

**Friday, March 11.**—Royal College of Surgeons: Professor C. Bolton's lecture on the "Pathology of Gastric Ulcer," 5.

Annual meeting, Polyclinic, 5.15.

General meeting, Royal Society of Medicine, 5.

Annual meeting, London Homœopathic Hospital, Great Ormond Street, 3.30.

